# Oral health in professional Slovak soccer players: Assessment of dental risks, subgingival microbiota and genetic influences

**DOI:** 10.1371/journal.pone.0351544

**Published:** 2026-06-18

**Authors:** Silvia Timkova, Veronika Martinska, Andrea Madarasova Geckova, Vladimir Elias, Michal Konecny, Peter Kizek

**Affiliations:** 1 Department of Stomatology, P. J. Šafárik University, L. P. University Hospital in Košice, Košice, Slovakia; 2 Periodental Ltd., Košice, Slovakia; 3 Department of Stomatology and Maxillofacial Surgery, P. J. Šafárik University, L. P. University Hospital in Košice, Košice, Slovakia; 4 Department of Health Psychology and Research Methodology, Faculty of Medicine, P. J. Šafárik University, Košice, Slovakia; 5 Department of Community and Occupational Medicine, University Medical Center Groningen, University of Groningen, Groningen, Netherlands; 6 Institute of Social Health at Palacký University Olomouc (OUSHI), Sts Cyril and Methodius Faculty of Theology, Olomouc, Czech Republic; 7 Laboratory of Genomic Medicine, AGEL Gen Ltd., Comenius University Science Park, Bratislava, Slovakia; 8 Department of Biology, Institute of Biology and Biotechnology, University of St. Cyril and Methodius in Trnava, Trnava, Slovakia; 9 Department of Genetics, Faculty of Natural Sciences, Comenius University Bratislava, Bratislava, Slovakia; Mae Fah Luang University School of Anti Aging and Regenerative Medicine, THAILAND

## Abstract

**Background:**

Recent studies reveal high rates of dental issues among professional soccer players, worsened by poor hygiene, frequent sport drinks consumption and limited preventive care. Busy schedules, frequent relocations and changing clubs further disrupt dental routines, impacting performance and well-being. Therefore, we decided to assess the oral health status of professional Slovak soccer players and address these critical concerns.

**Methods:**

We assessed the oral health of 51 male soccer players from two elite Slovak soccer clubs during 2023/2024 season. Data collection included two paper-based questionnaires and a clinical oral examination by dentists. Additionally, clinical laboratory data were collected from saliva to test for presence of periopathogenic bacteria and DNA polymorphisms.

**Results:**

Although 92.2% had valid health insurance, 36% did not attend dental check-ups in the past year, indicating underutilization of preventive care. While 86.2% brushed their teeth more than twice daily, only 48% practiced interdental cleaning and 35.3% used mouthwash. A significant 83.7% consumed sports drinks high in sugar and acid, influencing oral health risks. Clinical examinations revealed that 86.3% had a moderate to high DMF index (mean decayed teeth: 3.8) and 54.9% exhibited gingivitis. Bacterial analyses showed 25.5–74.5% carried highly periopathogenic bacteria, indicating a high risk for periodontitis. Additionally, 15.7% of players exhibited presence of DNA polymorphisms associated with risk of periodontitis onset.

**Conclusion:**

This study reveals a gap in preventive dental care among professional soccer players, emphasizing the urgent need for integrated oral health strategies within sports programs.

## Introduction

Soccer players are often perceived as the ideal of physical health, owing to their rigorous training regimens, high levels of physical activity and disciplined lifestyles. Their public image characterized by fitness, vitality and discipline reinforces the belief that they enjoy optimal health. But is this truly the case? How well do these athletes take care of their oral health? And more importantly, could their dental care routines (or lack thereof) potentially influence their performance on the field?

The connection between oral health and general well-being is well-documented, with poor dental hygiene contributing to systemic inflammation that may negatively affect both health and athletic performance. Dental issues such as cavities, gum disease and infections can lead to pain, discomfort and psychological stress – factors that could potentially impair an athlete’s ability to train and compete at their performance peak. Research by Solleveld et al. suggests that soccer players with compromised oral health may be more prone to injuries, indicating that dental conditions may act as underrecognized risk factors for athletic performance [1]. In addition, Needleman et al. found that over 40% of professional athletes had untreated dental problems, further suggesting a possible link between oral health to reduced performance [2]. Other studies expanded on this by demonstrating that dental conditions like caries and periodontitis contribute to the release of pro-inflammatory cytokines in response to bacterial infection, which may exacerbate muscle fatigue and increase injury risk [3–6]. For instance, individuals with the HLA-DRB104* allele may have immune cells that are more likely to produce higher levels of these cytokines when faced with bacterial infections [7]. Moreover, Bramantoro et al. (2020) highlighted that neglected oral hygiene in professional athletes is associated with decreased well-being and could potentially reduce performance by up to 18% [8, 9]. Chronic oral inflammation, particularly in high-stress environments where cortisol levels are elevated and immune systems are compromised, may undermine both training and competition outcomes [10].

In addition to the physical toll of poor oral health, the nutritional habits of soccer players further complicate their dental well-being. The frequent consumption of sugar-laden sports drinks, energy bars and gels, common in high-intensity sports, is linked to an increased risk of dental decay [9,11]. When combined with poor oral hygiene practices, dehydration and oxidative stress, these dietary habits may accelerate dental erosion, cavities and periodontal disease [9]. Despite being frequently overlooked, oral health is a critical component of overall well-being and its management could play a role in supporting athletes’ health and potentially their performance.

In Slovakia, professional soccer players are entitled to partially covered dental care through the national health insurance system upon signing a club contract. They are eligible for one preventive dental check-up per year, covered by the insurance company. Regular attendance also qualifies them for coverage of additional dental treatments. However, the frequent transfers between clubs, relocations and irregular dental follow-ups may heighten the risk of dental health issues within this population. The key questions remain: Are Slovak soccer players fully utilizing the dental services available to them? And are they aware of the potential impact that their oral health could have on both their well-being and athletic performance? Despite the expanding the international research on athletes’ oral health, comprehensive studies focusing specifically on professional soccer players in Slovakia remain scarce. Addressing this gap is crucial for the development of targeted preventive strategies and for a deeper understanding of the multifactorial determinants of oral health in this population. Although previous research has consistently reported a high prevalence of oral diseases among athletes, detailed insights into the composition of oral microbiota in professional soccer players—and its association with clinical oral health status—are still limited. The present study seeks to bridge this gap by integrating microbiological, clinical and behavioral data.

The aim of this study is to evaluate the oral health status of Slovak professional soccer players, assess their utilization of preventive dental care services and investigate how oral health problems could potentially relate to aspects of athletic performance, acknowledging that this study did not directly measure performance outcomes. Additionally, we aim to explore potential role of dietary habits, oral hygiene practices and genetic and bacterial risk factors in the development of oral diseases within this group. Through this research, we hope to raise awareness of the critical link between oral health and athletic performance and propose integrating regular oral health assessments into the routine medical care of soccer players.

## Materials and methods

### Sample and procedures

This cross-sectional study investigated the oral health of professional soccer players from two Slovak elite soccer clubs. The selection of the two Slovak elite soccer clubs was based on convenience sampling, primarily determined by accessibility and willingness of the clubs to participate. Both clubs compete at the highest national level, representing a relatively homogeneous group in terms of training intensity and professional status. The research was conducted in the season 2023/24 with players facilitated via the respective clubs (17 April 2023–19 May 2024). Two football clubs were approached, comprising a total of 52 participants. Of these, one participant – team coach – was excluded as he was not active player; therefore, the final analyzed cohort consisted of 51 active players. None of the included players reported systemic antibiotic use within the preceding 3 months. This information was obtained as part of the medical history prior to biological sampling. Data collection for this study involved several components. First, two paper-based questionnaires were administered directly to the soccer players. Prior to data collection, they were pilot-tested on a small group to ensure clarity and comprehensibility. Formal validation and reliability testing were not performed. The first questionnaire “*Personal history*” was designed to gather detailed background information about each player. The second questionnaire “*Dental care and risk factors*” aimed to assess the players´ dental care and hygiene practices and identify any potential risk factors. In addition, a third paper-based questionnaire was completed by dentists. This questionnaire focused on *“Clinical examination of oral health”* documented the dentist´s assessment of the players´ dental health. Clinical oral health assessment included evaluation of DMF (Decayed, Missing, Filled teeth) and BOP (Bleeding On Probing) indices. Prior to data collection, examiner calibration was performed to ensure consistency and reproducibility of clinical measurements. Clinical examinations were conducted by two experienced dentists. Periodontal probing was performed using a standardized six-site per tooth protocol (mesiobuccal, buccal, distobuccal, mesiolingual, lingual, distolingual). A standardized WHO periodontal probe (WHO/CPI probe) with a ball-ended tip (0.5 mm) and markings at 3.5–5.5 mm, 8.5 mm, and 11.5 mm was used. Probing was performed using a gentle, controlled force of approximately 20–25 g. BOP was assessed for each tooth; the examination was performed systematically by quadrants, and bleeding was recorded 10 seconds after probing each sextant. Furthermore, clinical laboratory data were collected by dentists to complement the questionnaire responses. A completed laboratory request form together with participant´s signed informed consent was completed and sent to Laboratory of Genomic Medicine, GHC GENETICS SK Ltd. (later renamed as AGEL Gen Ltd.). Microbiological assessment (PCR) was performed using a multisite sampling protocol, with samples collected from four sites, one per quadrant. Samples were obtained from the gingival sulcus at a depth of approximately 2–3 mm using sterile paper points, allowing absorption of crevicular fluid along with the present bacteria. No professional tooth cleaning was performed prior to sampling. Participants were instructed to brush their teeth in the morning on the day of examination without the use of toothpaste to minimize the influence of external chemical factors on microbiological findings. For each soccer player, multiple samples were taken from five different teeth to ensure the representativeness of the microbial sample. The laboratory analysis included DNA tests for periopathogenic bacteria and genetic variations represented by SNP polymorphisms in gene *HLA-DRB1*, which provided insights into the types and levels of bacteria present and genetic predispositions that might impact oral health. While laboratory testers were aware of player identity due to sample labeling for administrative purposes, they were blinded to the players’ clinical oral health scores (DMF/BOP). Data analysis was conducted independently to ensure objectivity and transparency regarding potential conflicts of interest. Paper-based questionnaires are presented in S1 File **Appendix A**.

### Ethical approval and consent to participate

The study was approved by the Ethics Committee of the Košice self-governing region (4243/2023/ODDZ-11835). All data and information gathered from the documentation, including demographic and clinical data, were used in accordance with the ethical standards as laid down in the 1964 Declaration of Helsinki and its later amendments or comparable ethical standards. Written informed consent to participate in this study was provided by all participants.

### Measures

Measures regarded personal history; dental care and risk factors; clinical oral examination by dentists; and laboratory DNA analysis. Questions are shown, in full, in **S1 File** **Appendix A**.

#### Background characteristics.

Background characteristics regarded age, gender, soccer club affiliation, playing position and health insurance status.

#### Personal history.

Personal history included information on the presence of diseases/injuries in the last 2 years, the presence of allergies and the use of any medication.

#### Dental care and risk factors.

Regarding **dental care,** players were asked about their dental visits over the past 12 months, including whether they had seen a dentist for issues such as preventive check-up, tooth decay (caries) or tooth extraction. Additionally, information was collected on visits to a dental hygienist within the same period. The study also inquired about players’ oral hygiene practices, specifically their frequency of tooth brushing (with an emphasis on brushing twice daily), regular interdental cleaning (at least once daily use of interdental aids) and regular mouthwash use (at least once daily). Regarding **risk factors**, several potential risk factors were evaluated. Players reported the presence of bleeding gums, bad breath and tooth sensitivity to cold or hot stimuli. The study also assessed lifestyle factors, including the consumption of sports drinks, current smoking habits and the occurrence of bruxism (teeth grinding).

#### Clinical examination of oral health.

Clinical examination of oral health consisted of clinical oral examination by dentists, laboratory DNA tests aimed for periopathogenic bacteria and genetic factor, represented by DNA polymorphism in *HLA-DRB1* gene, especially presence of risk allele *04. **A. Clinical oral examination by dentists** included a comprehensive assessment of both periodontal health and tooth conditions. Several indices were utilized to provide a standardized assessment of oral health, such as DMF index and BOP. DMF index was calculated for 28 teeth, and the results were categorized into three groups: low (DMF ≤ 3), moderate (DMF 4–9) and high (DMF > 10). For BOP, the most recent classification was applied, categorizing players based on the percentage of bleeding sites: no gingivitis (BOP% < 10%), localized gingivitis (BOP% ≥ 10% and ≤ 30%), or generalized gingivitis (BOP% > 30%) [12–14].

Before, DNA was isolated using innuPREP Forensic Kit (iST Innuscreen GmbH) and quantified using Invitrogen™ Qubit™ 1X dsDNA High Sensitivity Assay Kit (ThermoFisher) and Invitrogen™ Qubit™ 2.0 Fluorometer (ThermoFisher).

**B. Laboratory periopathogenic bacterial examination** aimed to identify bacterial factors contributing to periodontal disease. The examination detected 12 key periodontal pathogens categorized by pathogenicity to ***highly pathogenic*** *purple complex Aggregatibacter actinomycetemcomitans (A. actinomycetemcomitans); red complex Porphyromonas gingivalis (P. gingivalis), Treponema denticola (T. denticola), Tannerella forsythia (T. forsythia)* and *Filifactor alocis (F. alocis);* ***moderately pathogenic*** *orange and orange associated complex Prevotella intermedia (P. intermedia), Parvimonas micra (P. micra), Fusobacterium nucleatum (F. nucleatum), Campylobacter rectus (C. rectus) and Eubacterium nodatum (E. nodatum);* and ***low pathogenic*** *green complex Eikenella corrodens (E. corrodens), Capnocytophaga gingivalis (C. gingivalis)* [15]. The detection was performed using Real-Time PCR method with TaqMan probes for the 16S, 23S rRNA and *groL* gene of previously described bacteria on EliGene® Dental MultiBact Kit RT (Elisabeth Pharmacon). The C_T_ value for each detected probe was analysed, while the detection limit evaluated by the manufacturer was below 25 cycles. Lower C_T_ value corresponds to a higher frequency/concentration of the respective bacterial species in the sample. As a control of the procedure positive commercial control in the kit (PC DNA) and total bacteria (TB) content mix with the probe for general sequence of bacterial 16S rRNA gene was used. The Ct values were interpreted as follows: “−” (Ct > 25), “+” (Ct = 20–25), “++” (Ct = 15–20), and “+++” (Ct < 15), with lower Ct values indicating higher bacterial concentration. Based on the combination of detected pathogenic species and their respective Ct values, bacterial risk was classified as low (1), medium (2/3), or high (3 + /3). This classification was then used to summarize the overall periopathogenic risk among participants. The definition of the risk profile was based on classification proposed by Socransky et al. A high-risk profile was assigned when at least two bacteria from the red bacterial complex were detected, or the purple complex alone, regardless of the presence of other bacteria. A medium-risk profile was defined when one bacteria from the red complex was detected, or at least two bacteria from the orange complex, regardless of the presence of other bacteria. A low-risk profile was assigned when one bacteria from the orange complex was detected, or only bacteria from the orange aggregated or green complex were detected [16].

**C. Laboratory DNA variant examination** aimed to identify genetic predisposition related to the risk of developing periodontal disease. The DNA test was focused on detecting DNA polymorphisms in *HLA-DRB1* gene, especially risk allele * 04, using Real-Time PCR approach with EliGene® Coeliac RT kit (Elisabeth Pharmacon Ltd.) – CELI-DR4 Mix [17]. The C_T_ value for detected probes was analysed, while the detection limit evaluated by the manufacturer was determined below 35 cycles. For the control of the procedure the positive commercial control of the SYPL2 gene as a part of the kit (PC CELI Mix) was used.

### Statistical analysis

Descriptive statistics were used to summarize all data. Measures of central tendency (mean, median) and dispersion (standard deviation, range) were calculated for continuous variables. Frequencies and percentages were computed for categorical variables. Cross-tabulations and charts were employed to explore and visualize relationships between variables. Data were analysed using IBM SPSS Statistics 23 for Windows, and no inferential statistical tests were conducted.

## Results

### Background Characteristics

Our study sample consisted of 51 male soccer players from two elite sports clubs, representing various playing positions including goalkeepers, strikers, defenders and midfielders (11.8–33.3%). The mean age of the participants was 26.02/ ± 5.5, with a minimum age of 18 and a maximum age of 40 years. Regarding healthcare coverage, the majority of the players confirmed possessing valid health insurance in Slovakia, only four players reported the lack of such coverage, and other two reported being insured in another country. This suggests that the soccer players are likely in the peak of their physical health, with the majority having valid health insurance, which enables them to access preventive dental check-ups at no cost.

### Personal history

The majority of soccer players (66.7%) reported a history of various injuries mainly of the upper and lower limbs, while significant diseases were rare, with only a few cases of celiac disease in the last 2 years. Allergies and medication use were noted in a smaller proportion of players (27.3–22.2%), with medication primarily being used to manage allergies. The data suggests that while soccer players are prone to injuries due to nature of their sport, they generally enjoy good overall health, with only a minority reporting significant disease, allergies or medication use. For more details, see **Table 1**.

**Table 1:** Personal history of each soccer player (Questionnare No.1).

Variables	N (%)
**Presence of disease** ^1^	2 (4.4)
**Presence of injury** ^1^	30 (66.7)
**Presence of allergies** ^1^	12 (27.3)
**Using some medication** ^1^	10 (22.2)

^1^yes

### Dental care and risk factors

Regarding dental care, most players visited dentists in the last 12 months (64%), primarily for preventive check-ups, including assessment for decay or extractions. However, only a small proportion had also seen dental hygienists within the same period (15.7%). While most reported brushing their teeth more than once per day (86.2%), interdental cleaning (48%) and mouthwash use were less frequent (35.3%).

In terms of risk factors, a large proportion of the players regularly consumed sports drinks (83.7%), which have high sugar and acid content, potentially increasing the risk of tooth decay. Every third player reported experiencing tooth sensitivity (33.3%), which may indicate enamel erosion or decay. Additionally, 29.4% of players acknowledged the presence of bleeding gums, a potential sign of gum disease, and bad breath (19.6%), which could signal oral infections or poor hygiene. Smoking, as well as bruxism (teeth grinding), known to cause tooth wear and jaw pain, were also reported by a portion of the group (15.7−12%).

The findings suggest that although preventive dental check-ups are free of charge, 36% of soccer players still do not attend them, and the use of more specialized dental hygiene practices is also less frequent. Despite regular dental visits and good brushing habits, the players remain at significant risk for oral health issues. The insufficient use of interdental cleaning and mouthwash, combined with 83.7% consumption of sports drinks, as well as the prevalence of tooth sensitivity, bleeding gums and other risk factors, highlight the need to integrate preventive check-ups into the onboarding process for new players and include them in regular health assessment for all soccer players. For more details, see **Table 2**.

**Table 2:** Dental care and risk factors of each soccer player (Questionnaire No.2).

Variables	N (%)
***Dental care***	
**Visiting dentists in the last 12 months** ^1^	32 (64.0)
Reason – preventive check-up ^1^	22 (52.4)
Reason – tooth decay (caries) ^1^	12 (28.6)
Reason – tooth extraction^1^	5 (11.9)
**Visiting dental hygienist in last 12 months** ^1^	8 (15.7)
**Regular tooth brushing (twice/three times a day)** ^1^	44 (86.2)
**Regular interdental cleaning** ^1^	24 (48.0)
**Regular use of mouthwash** ^1^	18 (35.3)
***Risk factors***	
**Presence of bleeding gums** ^1^	15 (29.4)
**Presence of bad breath** ^1^	10 (19.6)
**Presence of tooth sensitivity (cold/hot)** ^1^	17 (33.3)
**Consumption of sports drinks** ^1^	41 (83.7)
**Smoking habit** ^1^	8 (15.7)
**Bruxism (teeth grinding)** ^1^	6 (12.0)

^1^yes

### Clinical examination of oral health

#### A. Clinical oral examination by dentists.

The clinical oral examination revealed several key findings related to gingival and dental health. In terms of dental health, 86.3% of the soccer players exhibited a moderate to high DMF index (DMF>3), indicating a significant prevalence of dental caries and poor dental health. The mean of decayed teeth was 3.8, with a range from 0 to 12. The mean of filled teeth was 6.4, ranging from 0–20. Missing teeth varied between 0 and 5, with 62.7% of players had no extractions. Additionally, around 54.9% of players presented with localized or generalized gingivitis, affecting 10% and more of the dental sites. These clinical results suggest that the soccer players in the study exhibited significant oral health issues, related to gum and periodontal health and tooth sensitivity, which may confirm their previously reported questionnaire responses. For more details, see **Table 3**.

**Table 3:** Clinical oral examination by dentists.

Variables	N (%)
**DMF index**	
Low ≤ 3	7 (13.7)
Moderate 4–9	19 (37.3)
High ≥ 10	25 (49.0)
**BOP index**	
no gingivitis (<10%)	23 (45.1)
localized gingivitis (≥10% – ≤30%)	26 (51.0)
generalized gingivitis (>30%)	2 (3.9)

#### B. Laboratory periopathogenic bacterial examination.

The bacterial analysis revealed the presence of both highly and moderately pathogenic types of bacteria, as well as less pathogenic strains in the studied sample, multiple types of bacteria were represented in each sample. Among the **highly pathogenic bacteria***, T. denticola* and *T. forsythia* were the most prevalent, both detected in 74.5% of the players. *P. gingivalis* was found in 25.5% of the soccer players, while *A. actinomycetemcomitans* was detected in 43.1%. **Moderately pathogenic bacteria** were also relatively common, with *F. nucleatum* detected in all soccer players (100%), and *P. micra* in 82.4%. Among the **low pathogenic types**, *E. corrodens* showed the highest prevalence at 92.2%. This distribution suggests a significant bacterial load of both highly and moderately pathogenic species in the oral microbiota, suggesting a heighten risk for development of periodontitis. This aligns with our previous clinical oral examination by dentists, that more than 50% of soccer players exhibited localized or generalized gingivitis, which, if left untreated, could progress to periodontitis. Therefore, the results may highlight a high risk for periodontitis development within this group. For more details, see **Table 4**.

**Table 4:** Laboratory periopathogenic bacterial examination.

Variables	N (%)
**Detection of (periopathogenic) bacterial profile for periodontitis**	
Low risk	6 (11.8)
Medium risk	6 (11.8)
High risk	39 (76.5)
**Highly pathogenic types of bacteria**	
*A. actinomycetemcomitans*	22 (43.1)
*F. alocis*	29 (56.9)
*P. gingivalis*	13 (25.5)
*T. denticola*	38 (74.5)
*T. forsythia*	38 (74.5)
**Moderately pathogenic types of bacteria**	
*P. intermedia*	31 (60.8)
*P. micra*	42 (82.4)
*F. nucleatum*	51 (100.0)
*C. rectus*	35 (68.6)
*E. nodatum*	19 (37.3)
**Low pathogenic types of bacteria**	
*E. corrodens*	47 (92.2)
*C. gingivalis*	41 (80.4)

#### C. Laboratory DNA variant examination.

The genetic DNA analysis identified the presence of the HLA-DRB1*04 allele in 8 of soccer players, indicating prevalence of approximately 15.7% within this athlete population. The significance of this finding lies in the association of the HLA-DRB1*04 allele with potential periodontal disease risk. While this allele has been associated with various autoimmune disease in the literature, our study did not assess clinical autoimmune outcomes. Together, these findings may reinforce the high risk of periodontitis development in our group of soccer players. For more details, see **Table 5**.

**Table 5:** Laboratory DNA variant examination.

Variables	N (%)
**Detection of DNA polymorphism for periodontitis**	
Presence of risk allele HLA-DRB1*04	8 (15.7)

## Discussion

### 1. Prevalence of oral health issues despite free access to care

Our study revealed that 36% of soccer players did not attend dental check-ups, despite being eligible for one preventive dental check-up covered by Slovakia’s national health insurance. While 86.2% of the players reported brushing their teeth more than once a day, less than half (48%) engaged in interdental cleaning, and only 35.3% used mouthwash. This finding aligns with Needleman et al., who found that professional athletes often overlook regular dental check-ups, leading to untreated dental issues [18]. Similarly, Gay-Escoda et al. identified that access to healthcare does not necessarily translate into its utilization, as many athletes underestimate the importance of preventive care [19]. Solleveld et al. further emphasized the need for increased awareness among athletes regarding oral health, even when healthcare services are readily accessible [1]. This gap between access and behaviour indicates a pattern in our data, highlighting that providing free care may not be sufficient; targeted educational interventions that emphasize the importance of regular dental visits and advanced hygiene practices may be needed. From this, our findings suggest that behavioural factors may play a crucial role in oral health outcomes, even in populations with easy access to care.

### 2. High risk of dental caries and periodontal disease

We observed a significant prevalence of dental caries and periodontal disease, with 86.3% of soccer players showing a moderate to high DMF index and 54.9% exhibiting gingivitis. These findings are in line with studies, which found that professional athletes are susceptible to periodontal disease due to a combination of high sugar intake and inadequate oral hygiene [20–22]. Similarly, other studies reported high levels of dental caries among athletes, often attributed to poor dietary habits and insufficient oral care routines [9,23]. This is further supported by Ashley et al., who noted that athletes in endurance sports, such as soccer, face a higher risk of oral health problems, exacerbated by dehydration and reduced salivary flow during intense physical activity [24]. This findings suggest a possible relationship between hygiene practices, dietary factors and the observed prevalence of dental caries and periodontal disease in soccer players, similar to trends reported in other elite athletes.

### 3. Diet and its contribution to oral health decline

Our study found that 83.7% of soccer players regularly consumed sugar-laden sports drinks, contributing to increased risk of dental erosion and decay. Additionally, 33.3% reported tooth sensitivity, and 29.4% experienced bleeding gums – common signs of enamel erosion and periodontal inflammation. These results align with the findings of other studies, which demonstrated the role of sports drinks in exacerbating tooth decay among athletes [25,11]. Schulze et al. similarly highlighted the connection between frequent consumption of energy supplements and dental health issues, pointing out that many athletes are unaware of the cumulative effects of sugar and acid on their teeth. Poor dietary habits in athletes, combined with inadequate hydration, accelerate enamel erosion, particularly in those who do not maintain comprehensive oral hygiene routines [9]. Our data reveal pattern suggesting that nutritional habits may be associated with oral health outcomes. These results highlight the potential benefit of integrating nutritional counselling with oral hygiene education, raising awareness of the long-term impact of dietary habits.

### 4. Possible bacterial and genetic susceptibility for periodontitis

Bacterial and genetic DNA analyses revealed that 25.5–74.5% of soccer players carried highly pathogenic bacteria, including key periodontal pathogens *P. gingivalis* and *A. actinomycetemcomitans*. Additionally, 15.7% of players exhibited genetic predispositions for periodontitis and among these 25–62.5% also hosted highly pathogenic bacteria. Although research specifically examining these pathogens in athletes is limited, existing studies suggest significant implications for oral health and overall well-being. The high bacterial levels of *P. gingivalis* and *A. actinomycetemcomitans*, both late colonizers in periodontal disease, raise serious health concerns. *P. gingivalis* possesses virulence factors like collagenases and proteases, that drive tissue destruction and was found in 85.8% of chronic periodontitis samples [26,27]. Its interactions with early colonizers form a pathogenic complex linked to systemic conditions, including Alzheimer’s disease, atherosclerosis, insulin resistance in diabetes and certain cancers [28–36].

Similarly, *A. actinomycetemcomitans* is linked to periodontal and systemic diseases due to generation of virulence factors, such as leukotoxin A and lipopolysaccharides [35,37]. It is associated with worse periodontal outcomes in anabolic steroid users, who show greater probing depths and higher severe periodontitis prevalence [38]. Additionally, elevated levels of *A. actinomycetemcomitans* in diabetic nephropathy patients correlate with increased cerebral infarction incidence and oral health decline [39]. In chronic kidney disease, this bacterium promotes inflammation and oxidative stress, exacerbating immune inefficiencies and it has been implicated in rheumatoid arthritis [37,40,41]. Moreover, *A. actinomycetemcomitans* can cause empyema necessitans following oral trauma or infection [42]. Its immune-evasion capabilities contribute to tissue damage and progressive bone loss in periodontitis, with microbial profiles revealing a unique immune imbalance in aggressive periodontitis marked by lower immunoglobulin G and interleukin-10 levels [43].

The HLA-DRB1*04 allele is a significant genetic marker within the Human Leukocyte Antigen (HLA) system, particularly in the major histocompatibility complex (MHC) class II genes, which are essential for the immune system. The *HLA-DRB1* gene encodes a protein critical for this process and includes several subtypes, such as HLA-DRB1*0401 and HLA-DRB1*0402, which can influence disease susceptibility differently. Presence of HLA-DRB1*04 is associated with several autoimmune conditions, including rheumatoid arthritis, systemic lupus erythematosus, celiac disease and multiple sclerosis [44–49]. Importantly, it has been positively linked to aggressive periodontitis through specific haplotypes, indicating that individuals with this allele may have a higher risk of this form of periodontal disease [50,51]. In our study, 8 out of 51 professional soccer players carry the HLA-DRB1*04 allele, suggesting a potential genetic predisposition to both autoimmune conditions and periodontal disease. Our findings suggest a possible bacterial and genetic susceptibility to the onset of periodontitis among professional soccer players.

### 5. Oral health as a performance factor

Our study supports the potential association between oral health and aspects of athletic performance. While we did not directly measure injury rates, muscle fatigue, or athletic performance outcomes, the data indicated trends consistent with previous literature suggesting that oral health may be related to performance, recovery and injury susceptibility. Consistent with Solleveld et al., oral diseases like caries and periodontitis may exacerbate inflammation, which could lead to greater injury susceptibility [1]. Needleman et al. similarly reported that 40% of professional athletes experience oral health issues, which could potentially reduce performance due to pain, stress and systemic effects like inflammation [2,17]. Gay-Escoda et al. also demonstrated that untreated oral infections and inflammation may impair training and recovery by increasing systemic inflammation and muscle fatigue [18]. This is further supported by studies, which reported that oral health-related pain could reduce athletes’ focus and endurance [10,18]. We learned that oral health management should be regarded as an integral part of an athlete’s overall health strategy, as it may affects physical performance, recovery and injury risk, while still acknowledging that we did not measure performance outcomes directly.

#### 6. Oral health neglect as a hidden risk factor in professional soccer.

Our findings suggest that oral health is an underappreciated aspect of health management in professional soccer players, despite its potential to affect both health and performance. The high prevalence of dental decay, gingivitis and bacterial infections, coupled with frequent club transfers and inconsistent follow-ups, indicates a gap in the continuity of care. This finding aligns with Gay-Escoda et al., who observed that many athletes neglect oral health, often due to the logistical challenges of maintaining consistent care during frequent relocations [19]. Ashley et al. also highlighted the need for sports organizations to integrate oral health into their broader health strategies, as untreated dental issues can have cumulative long-term effects on athletes’ well-being [24]. This view is further supported by Stamos et al., who advocate for a universal protocol for dental examinations in athletes to ensure more consistent and proactive oral health management [52]. Our findings reveal a notable trend indicating that consistent oral health evaluations during transfers and relocations may be important for maintaining overall health in professional athletes. These results suggest a possible relationship between neglected dental care and long-term health risks in this population.

### Strengths and limitations

This study has several strengths and limitations. One of its strengths is the comprehensive assessment approach, utilizing questionnaires, clinical examinations and laboratory analyses, which provides a thorough understanding of the soccer players’ oral health status. Additionally, the focus on Slovak professional soccer players addresses a unique demographic that has been under-researched regarding oral health issues. Moreover, the inclusion of genetic and bacterial analyses offers insights into the multifactorial nature of oral health risks among athletes and the findings have practical implications for improving both oral health and may potentially support overall performance. However, the study is limited by its descriptive nature, which does not allow for causal inferences between oral health issues and athletic performance or other contributing factors. The lack of inferential statistical analyses limits our ability to formally assess associations between variables, and consequently, the conclusions regarding potential relationships remain more speculative than evidence-based. The reliance on self-reported data for dental habits may introduce bias, as players might overestimate their adherence to good oral hygiene practices. The study included a relatively small sample size of 51 professional male soccer players, which was determined by the accessible population in the two elite Slovak clubs during the 2023/2024 season. No formal power calculation was performed, as the study was designed as exploratory. Furthermore, the findings may not be generalizable to soccer players from different countries or levels of competition, given that cultural and environmental factors can influence oral health behaviours. The study also excludes female players, potentially overlooking differences in oral health issues and behaviours among female athletes. Only male participants were included, as no elite female soccer club was available at the time of data collection. The inclusion of female athletes represents an important direction for future research, which we plan to address in subsequent studies. Additionally, future research should address the lack of assessed performance indicators and injury rates by incorporating longitudinal designs, objective performance metrics, and injury surveillance data to better elucidate the potential relationship between oral health and athletic performance. Overall, while this descriptive study offers valuable insights into the oral health of professional soccer players, its limitations should be acknowledged when interpreting the results.

### Implications

Based on the findings, the implications are:

1. **Mandatory dental screenings** upon signing new contracts and periodic check-ups throughout the season.
2. **Oral health education programs & leaflets** spreading information about identified dental risks of soccer players (**S1 Fig**) integrated into athlete training to increase awareness of the impacts of oral hygiene and dietary habits. As part of this initiative, provide and distribute leaflets outlining essential habits for maintaining healthy teeth and gums, developed based on insights from our study.
3. **Nutritional interventions** focused on reducing sugar intake from sports drinks and promoting alternatives that support both hydration and oral health.
4. **Personalized care plans** for players identified as being at higher risk for periodontitis, based on genetic and bacterial screenings.
5. **Coordination between clubs** to ensure consistent access to dental care services, especially during transfers or relocations.

## Conclusions

This study highlights significant oral health challenges among professional soccer players, despite access to a preventive dental check-up covered by national health insurance. A considerable proportion of players exhibit poor dental hygiene practices, high rates of dental caries, periodontal disease and an increased risk for periodontitis, exacerbated by their dietary habits and genetic predispositions. The findings underscore the need for improved oral health education, routine screenings and targeted interventions to address both hygiene and dietary factors. Integrating oral health into regular health assessments for athletes is essential to enhance both their well-being and athletic performance. Protect your oral health as you protect your sport performance!

## Supporting information

S1 File Appendix A. Paper-based questionnaires.
(DOCX)

S1 Fig Dental risks of soccer players. (PNG)

S2 File Soccer_data_final. (XLSX)
