# Supplementary material for: Oral health in professional Slovak soccer players: Assessment of dental risks, subgingival microbiota and genetic influences
Source: PLoS One. 2026 Jun 18;21(6):e0351544. doi: 10.1371/journal.pone.0351544 (PMC13278445; doi:10.1371/journal.pone.0351544)
Supplement: S1 File — Paper-based questionnaires. (DOCX) [file pone.0351544.s001.docx]

## **Questionnaire 1 – Personal history of soccer player**

**Name and Surname:**

**Age:**

1. **Do you have any disease right now?**

☐ Yes ☐ No

1. **Have you had any injuries in the last 2 years?**

☐ Yes ☐ No

1. **Do you have any allergies?**

☐ Yes ☐ No

*If yes, please specify:* ______________________________

1. **Are you currently taking any medication?**

☐ Yes ☐ No

*If yes, please specify:* ______________________________

## **Questionnaire 2 – Dental care and risk factors of soccer players**

1. **Have you visited a dentist in the last 12 months?**

☐ Yes ☐ No

If yes, what was the reason?

☐ Preventive check-up

☐ Tooth decay

☐ Tooth extraction

1. **Have you visited a dental hygienist in the last 12 months?**

☐ Yes ☐ No

1. **How often do you clean your teeth?**

☐ Once daily

☐ Twice daily

☐ Three times daily

1. **Do you regularly clean your interdental spaces?**

☐ Yes ☐ No

1. **Do you use mouthwash?**

☐ Yes ☐ No

1. **Are your gums bleeding?**

☐ Yes ☐ No

1. **Do you have a bad breath?**

☐ Yes ☐ No

1. **Do you feel that your teeth are sensitive to cold or warm temperatures?**

☐ Yes ☐ No

1. **Do you drink energy or ionic drinks?**

☐ Yes ☐ No

1. **Do you smoke?**

☐ Yes ☐ No

1. **Do you grind your teeth?**

☐ Yes ☐ No
