# Supplementary figures and images for: Oral health in professional Slovak soccer players: Assessment of dental risks, subgingival microbiota and genetic influences
Source: PLoS One. 2026 Jun 18;21(6):e0351544. doi: 10.1371/journal.pone.0351544 (PMC13278445; doi:10.1371/journal.pone.0351544)

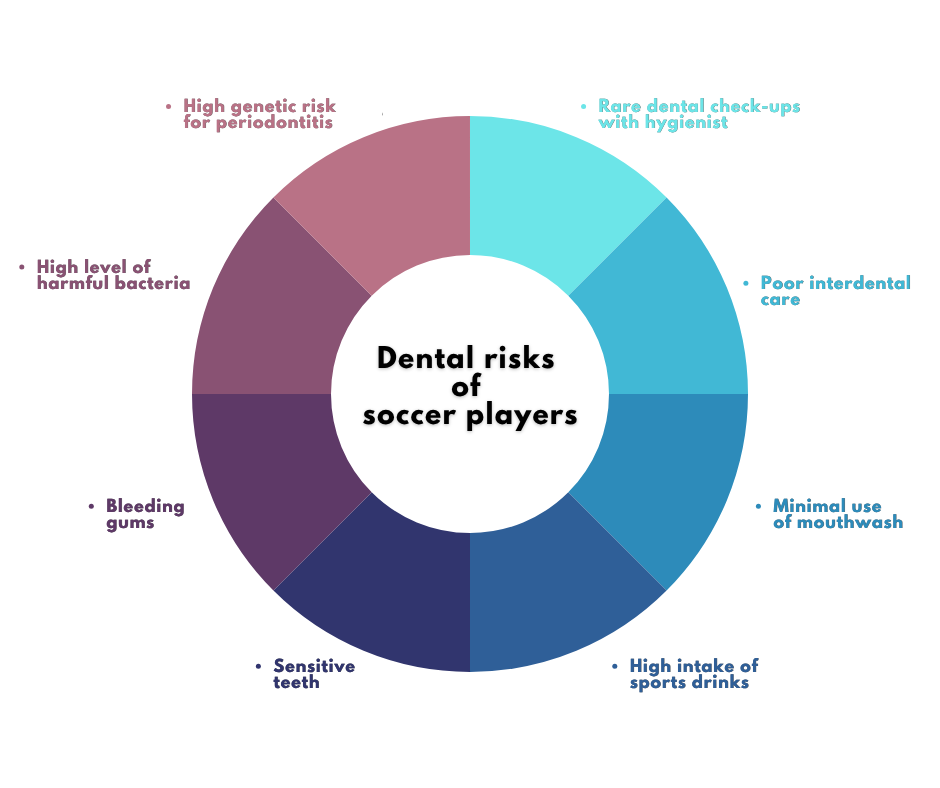

Supplement: S1 Fig — (PNG) [file pone.0351544.s002.png]
